# 

**Published:** 1882-12

**Authors:** 


CONTENTS.

| | PAGE. |
|---|---|
| Several Good Offers | 523 |
| Too Lean | 524 |
| Influence of the Mind Over the Body | 525 |
| How Valuable Lives are Lost | 526 |
| Bad Teeth and Disease | 527 |
| Soap and Soap Making | 527 |
| Nutrition in Health and Disease | 529 |
| Hunger and Appetite | 531 |
| Gems from Many Authors | 532 |
| The Decline of Faith (poetry) | 533 |
| The Collection of American Woods at the Central Park Museum | 534 |
| Ostrich Plucking in South Africa | 535 |
| Man and Animals | 536 |
| Poisonous Leaves | 537 |
| Comparison of English and American Railway Cars | 538 |
| Professor Haeckel's Life in Ceylon | 541 |
| Suggestions for a Family Ice-House | 542 |
| A Great Wrong Righted | 544 |
| Paragraphs | 540, 548 |

